# *Lactobacillus reuteri* FYNLJ109L1 Attenuating Metabolic Syndrome in Mice via Gut Microbiota Modulation and Alleviating Inflammation

**DOI:** 10.3390/foods10092081

**Published:** 2021-09-02

**Authors:** Bo Yang, Fuli Zheng, Catherine Stanton, Reynolds Paul Ross, Jianxin Zhao, Hao Zhang, Wei Chen

**Affiliations:** 1 State Key Laboratory of Food Science and Technology, Jiangnan University, Wuxi 214122, China; bo.yang@jiangnan.edu.cn (B.Y.); zhengfuli66@163.com (F.Z.); zhaojianxin@jiangnan.edu.cn (J.Z.); chenwei66@jiangnan.edu.cn (W.C.); 2 School of Food Science and Technology, Jiangnan University, Wuxi 214122, China; 3 International Joint Research Laboratory for Pharmabiotics & Antibiotic Resistance, Jiangnan University, Wuxi 214122, China; catherine.stanton@teagasc.ie (C.S.); p.ross@ucc.ie (R.P.R.); 4 APC Microbiome Ireland, University College Cork, T12 K8AF Cork, Ireland; 5 Teagasc Food Research Centre, Moorepark, Fermoy, P61 C996 Co. Cork, Ireland; 6 National Engineering Research Center for Functional Food, Jiangnan University, Wuxi 214122, China; 7 Wuxi Translational Medicine Research Center, Jiangsu Translational Medicine Research Institute Wuxi Branch, Wuxi 214122, China

**Keywords:** *Lactobacillus reuteri*, metabolic syndrome, gut microbiota

## Abstract

Metabolic syndrome is caused by an excessive energy intake in a long-term, high-fat and/or high-sugar diet, resulting in obesity and a series of related complications, which has become a global health concern. Probiotics intervention can regulate the gut microbiota and relieve the systemic and chronic low-grade inflammation, which is an alternative to relieving metabolic syndrome. The aim of this work was to explore the alleviation of two different *Lactobacillus* *reuteri* strains on metabolic syndrome. Between the two *L. reuteri* strains, FYNLJ109L1 had a better improvement effect on blood glucose, blood lipid, liver tissue damage and other related indexes than NCIMB 30242. In particular, FYNLJ109L1 reduced weight gain, food intake and fat accumulation. Additionally, it can regulate the gut microbiota, increase IL-10, and reduce IL-6 and tumor necrosis factor-α (TNF-α), as well as liver injury, and further reduce insulin resistance and regulate lipid metabolism disorders. In addition, it could modulate the gut microbiota, particularly a decreased *Romboutsia* and *Clostridium sensu stricto-1*, and an increased *Acetatifactor*. The results indicated that FYNLJ109L1 could improve metabolic syndrome significantly via alleviating inflammation and gut microbiota modulation.

## 1. Introduction

Metabolic syndrome (MS) is a clinical syndrome caused by obesity, impaired glucose regulation, hypertension, dyslipidemia and insulin resistance, leading to the occurrence and development of cardiovascular disease (CVD), hypertension, type 2 diabetes (T2D), and non-alcoholic fatty liver disease (NAFLD) [1], which has become a catastrophic epidemic globally [2]. MS prevalence is currently increasing in both developed and developing countries, with a statistically significant prevalence of 20–25% of MS in adult buckles worldwide [3]. Due to additional evidence, it is widely believed that dietary changes resulting in an excess nutrition could lead to MS. Different dietary structures could affect the weight difference and metabolism regulation directly, and can change the gut microbiota [4], for instance, mice fed with a high-fat diet (HFD) for a long time showed an imbalanced gut microbiota [5]. The decrease in health-promoting bacteria such as lactic acid bacteria and butyric acid producers and the increase in harmful bacteria such as pro-inflammatory and pathogenic bacteria are important reasons for the occurrence of chronic inflammation and metabolism disorders [6,7].

The main treatments for MS are lifestyle change and medication. However, lifestyle changes need to be maintained for a long time, and drug treatment, although effectively, may have undesirable side-effects, particularly liver damage [8]. Therefore, there is an urgent need for a safe and effective treatment that can alleviate MS. Notably, probiotics, which are considered safe and basically have no side effects, are considered to have a good intervention effect on a variety of diseases including MS [9]. *Lactobacillus* is generally recognized as safe and is one of the widely used probiotics for mammals [10]. It demonstrates a key role in regulating the host metabolism, and the active metabolites produced through metabolism have positive effects on the gut microbiota [11,12]. For example, *L. reuteri*, as a native resident of the human and animal gastrointestinal tracts, has been verified in animal experiments to reduce insulin resistance, the formation of hepatic steatosis, hyperlipidemia, along with the prevention of elevated blood sugar and related MS [13,14]. It was reported that different strains, even from the same species, had different effects on metabolic disorders. Mice intervened with *L. reuteri* ATCC PTA 4659 showed a significantly lower body weight than that in the control group, which was significantly increased in the *L. reuteri* L6798-treated group [15]. However, the relationship between the host gut microbiota, chronic inflammation, and metabolic disorders has not been adequately investigated with different *L. reuteri* intervention strategies. *L. reuteri* NCIMB 30,242 with a high BSH activity can affect the serum cholesterol in vitro, in rats, and in clinical trials [14,16], which is related to cardiovascular health; however, we presume that it might have potential benefits on metabolic disorders. In the current work, two *L. reuteri* strains FYNLJ109L1 and NCIMB 30,242 with the same origins, isolated from a piglet fecal sample, were used to investigate the remission effect of different *L. reuteri* strains on the MS induced by an HFD and were compared to explore the correlation between the gut microbiota, inflammation, and MS.

## 2. Materials and Methods

### 2.1. Bacterial Preparation

Two *L. reuteri* strains were used in this study, in which FYNLJ109L1, originally isolated from pig fecal samples in Lijiang, Yunnan Province, China, was deposited at the Collection Center of Food Microbiology (CCFM)-Jiangnan University, and *L. reuteri* NCIMB 30,242 was from a porcine origin and with cholesterol lowering capabilities [16,17]. The bacteria were sub-cultured three times in an MRS broth. Then the bacteria were collected via centrifugation at 6000× *g* for 15 min. The cell pellets were mixed with a cryoprotectant, which contained 13% skim milk, 2% trehalose and 2% sucrose, and lyophilized and stored at 4 °C.

### 2.2. Animals Experiment Design

The animal experiment was approved by the Animal Ethics Committee of Jiangnan University (JN. No20200630c1201130(121)), and the whole experimental process conformed to the Jiangsu Provincial Measures for the Control of Experimental Animals. Forty-eight C57BL/6J mice (5-week-old, male) without specific pathogens were obtained from the Institute of Model Zoology, Nanjing University (Nanjing, Jiangsu, China). Mice were raised in a specific pathogen-free (SPF) facility under standard conditions (constant temperature of 20 ± 2 °C, humidity of 50 ± 5%, and 12-hour light–dark cycle).

Mice were randomly divided into six groups (each *n* = 8), including one control group, one HFD group, two drug control groups (Simvastatin, Metformin) and two *L. reuteri* intervention groups (Table 1). The control mice were fed with a normal control diet (NC, total energy 3.6 kcal/g, 10% fat), and the other mice were fed with an HFD (total energy 5.0 kcal/g, 60% fat). During the period of diet induction, the two *L. reuteri* groups were given 0.2 mL of freeze-dried bacterial powder resuspended with physiological saline (10^9^ CFU), and the drug groups were administrated with simvastatin at 3 mg/(kg BW) and metformin at 150 mg/(kg BW), respectively. After one week of acclimatization, all the mice were gavaged 6 times each week, and their body weight and food intake were monitored weekly. The whole trial lasted for 14 weeks. An oral glucose tolerance test (OGTT) was carried out at the 13th week of the trial. The body function of the mice was basically restored, and fecal samples were obtained in 14th week. Then, the mice were sacrificed for blood and tissue collection. The liver tissues were immersed in 4% neutral paraformaldehyde solution for fixation, and stool samples and the rest of the tissue samples were frozen at −80 °C.

### 2.3. Serum Biochemical Analysis of Blood Lipid Contents

After the blood was left standing for 3 h, the serum was collected by centrifuging (1000× *g*, 15 min). Serum lipids were measured by automatic biochemical analyzer (SELECRTA-E, Vital Scientific, Van Selavig, Netherlands), including triacylglycerol (TG), total cholesterol (TC), low-density lipoprotein cholesterol (LDL-C) and high-density lipoprotein cholesterol (HDL-C). In addition, the ratio of LDL-C to HDL-C was calculated as the atherosclerosis index (AI).

### 2.4. Glucose and Insulin Tests

Fasting blood glucose at 0 h was measured by glucose meter (Roche, Mannheim, Germany) after the mice were fasted overnight. Then, 2 g/(kg BW) of glucose was administered to the mice, and the concentrations of blood glucose were measured 30, 60 and 120 min later, respectively. According to the calculation time and the area under the curve of blood glucose value (AUCglucose), a glucose tolerance value was obtained. The fasting serum insulin content in the mice was determined by a commercialized kit (Nanjing Senbeijia Biological Technology, Nanjing, Jiangsu, China). The homeostasis model assessment of insulin resistance was used to evaluate the insulin resistance, and the calculation was performed as follow: fasting blood glucose × fasting serum insulin/22.5.

### 2.5. Pathological Morphology of the Liver

Treatment of liver tissue was carried out according to a previous study [18]. The same part of each mouse liver sample was collected, fixed, dehydrated, embedded, cut into ~5 μm thick slices, and finally stained with H & E. The liver micrographs were evaluated by the SAF scoring system [19].

### 2.6. Determination of Cytokines in the Liver

Liver tissue samples of 150 mg were crushed with a buffer solution to prepare the liver homogenate, then centrifuged (10,000× *g*, 15 min, 4 °C) to collect the supernatant. Next, three cytokines (TNF-α, IL-10, IL-6) were determined by the commercialized ELISA kit (R&D Systems, Minneapolis, MN, USA) and the protein concentration was determined with a commercialized kit (Beyotime Biotechnology, Shanghai, China).

### 2.7. The Analysis of the SCFAs in Feces

Analysis of short chain fatty acids (SCFAs) were performed as previously described [20]. The feces stored at −80 °C were taken out, lyophilized, weighed and put into a sterile centrifuge tube, then mixed with 500 μL saturated sodium chloride solution and soaked for 30 min. Next, 40 μL of 10% (*v*/*v*) H_2_SO_4_ was added to acidify the samples to make them fully dissolved. After acidification, the samples were homogenized with 1 mL ethyl ether and centrifuged (4 °C, 13,000× *g*, 15 min). Then, the supernatant was removed to a clean tube with 0.25 g anhydrous sodium sulfate in advance and stood for 15 min to absorb water. After centrifugation and filtration, it was analysed by GC-MS (GCMS-QP2010 Ultra system, Shimadzu Corporation, Kyoto, Japan) as previously described [21].

### 2.8. Analysis of the Gut Microbiota

Fecal genomic DNA was extracted with FastDNA SPIN Kit for Feces (MP Biomedicals, Santa Ana, CA, USA), and then amplified, purified, quantified and mixed with equal quality samples [22]. Library preparation, library testing, and MISeq platform PE300 sequencing were performed as previously described [23]. After sequencing, the QIIME2 pipeline was used to analyze the deplaning data [24]. Linear discriminant analysis effect size (LEfSe) analyses were performed to identify the specific taxa on the MicrobiomeAnalyst online platform [25]. The indexess of Chao 1 and Simpson were used to reflect the alpha diversity of the gut microbiota. Additionally, the beta diversity of gut microbiota was assessed for differences in species composition using the principal coordinate analysis (PCoA) method and visualization [26].

### 2.9. Statistical Analysis

Data were presented using mean ± SEM. The statistical assessments among the six groups were calculated by a one-way ANOVA with Tukey’s post-hoc test (parametric analysis) or the Kruskal–Wallis test followed by Duncan’s post-hoc test (non-parametric analysis). A *p*-value < 0.05 was defined as statistically significant.

## 3. Results

### 3.1. Effect of L. reuteri on Body Weight, Food Intake and Fat Index

**Figure 1:**
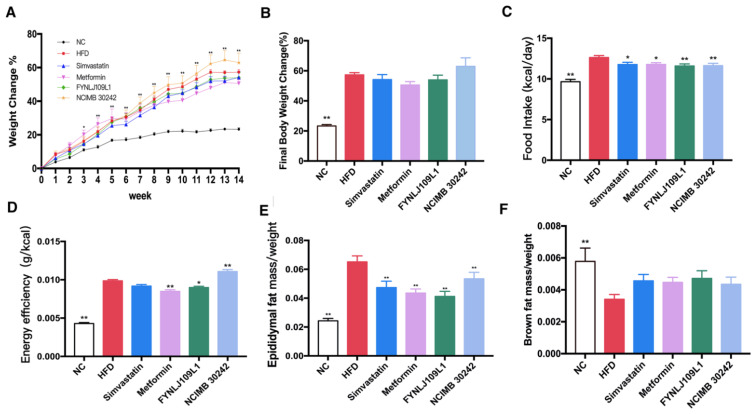
Effects of *L. reuteri* intervention on body weight, food intake and fat ratio. (**A**) Body weight. The *t*-test was only performed between NC and HFD, * *p* < 0.05 and ** *p* < 0.01; (**B**) Final body weight change; (**C**) Food intake; (**D**) Energy efficiency; (**E**) Adipose mass/weight ratio; (**F**) Ratio of brown fat to weight. (*n* = 8. * *p* < 0.05 and ** *p* < 0.01 for comparison with HFD).

The pathological feature of MS is central obesity, which is primarily manifested by changes in body weight, dietary intake and fat proportion. At the third week of the animal trial, body weight differences were significantly different between NC and HFD groups (*p* < 0.05), but not different in other groups (Figure 1A). Before ending the trial, the body weight of the mice which were fed with different diets in the HFD and NC groups increased by 57.30% and 23.33%, respectively (Figure 1B). In addition, the daily feed intake and energy conversion efficiency in the HFD-fed mice were higher than those in the NC mice during the whole experiment period, which also led to a higher proportion of epididymal fat and a lower proportion of brown fat in the HFD-fed mice (Figure 1C–F). After interventions with *L. reuteri* for 14 weeks, the food intake and energy conversion rate were significantly decreased (*p* < 0.05), and in particular, the epididymal fat proportion in the *L. reuteri* FYNLJ109L1 treated mice was significantly decreased (*p* < 0.01), whereas the brown fat index was increased but without significance.

### 3.2. Effect of L. reuteri on Serum Lipid, Glucose and Insulin

**Figure 2:**
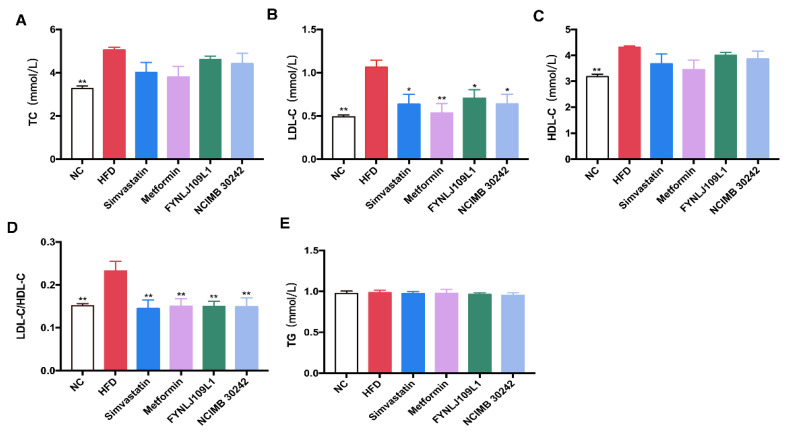
Effects of *L. reuteri* intervention on serum lipid levels. (**A**) TC; (**B**) LDL-C; (**C**) HDL-C; (**D**) LDL-C/HDL-C ratio; (**E**) TG. (*n* = 8. * *p* < 0.05 and ** *p* < 0.01 for comparison with HFD.).

Serum LDL-C, HDL-C, LDL-C/HDL-C and TC in the mice with a long-term intake of an HFD were significantly higher than those in the control mice (*p* < 0.01), but not for serum TG (Figure 2A–E). Serum LDL-C and LDL-C/HDL-C were significantly decreased after *L. reuteri* intervention (*p* < 0.01).

**Figure 3:**
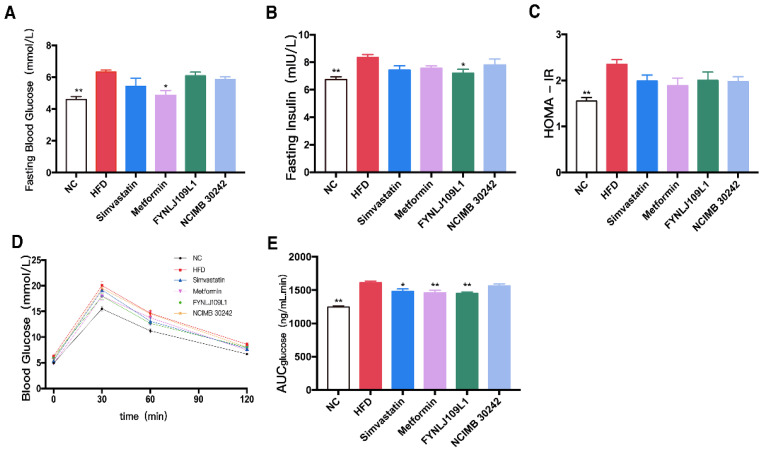
Effects of *L. reuteri* intervention on serum glucose and insulin levels. (**A**) Fasting blood glucose; (**B**) Fasting insulin; (**C**) HOMA-IR; (**D**) Blood glucose before and after the oral glucose; (**E**) AUC of the OGGT. NC, normal control; HFD, high-fat diet. Data are represented as mean ± SEM (*n* = 8). * *p* < 0.05 and ** *p* < 0.01 for comparison with HFD.

At the end of the experiment, high-fasting blood glucose (Figure 3A), high-fasting insulin (Figure 3B), the onset of insulin resistance (Figure 3C) and blood glucose recovered slowly after an OGTT (Figure 3D), and high AUCglucose values (Figure 3E) were found in the HFD group. *L. reuteri* FYNLJ109L1 treatment could moderately reduce the fasting insulin (*p* < 0.05), and significantly increase the clearance of glucose clusters during the OGTT period (*p* < 0.01), which was superior to *L. reuteri* NCIMB 30242.

### 3.3. Effect of L. reuteri on Liver Injury

**Figure 4:**
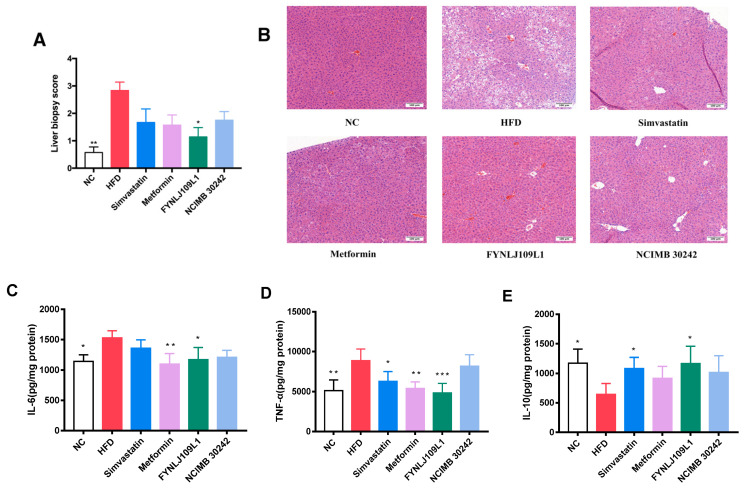
Effects of *L. reuteri* intervention on liver injury. (**A**) Liver tissue pathology score; (**B**) Representative pictures of H&E-stained liver sections from indicated mice (final magnification, 200×); (**C**) Liver IL-6 concentration; (**D**) Liver TNF-α concentration; (**E**) Liver IL-10 concentration.NC, normal control; HFD, high-fat diet. Data are represented as mean ± SEM (*n* = 8). * *p* < 0.05, ** *p* < 0.01 and *** *p* < 0.001 for comparison with HFD.

In the HFD group, the results were consistent with vacuolar degeneration of hepatocytes, the presence of lipid droplets accounting for 30–50% of the field of vision, and infiltration of inflammatory cells (Figure 4A,B). Regarding the level of liver inflammation, compared to the control mice, the concentrations of TNF-α and IL-6 in the HFD-fed mice were significantly increased, whereas the concentration of IL-10 was significantly decreased (*p* < 0.01) compared with that in the control group (Figure 4C–E). Histopathological scores showed that the intervention of *L. reuteri* FYNLJ109L1 significantly reduced the liver lesions (*p* < 0.01). In addition, compared with the HFD group, TNF-α (*p* < 0.001) and IL-6 (*p* < 0.01) in the *L. reuteri* FYNLJ109L1 treated mice decreased significantly, respectively. Moreover, the concentration of IL-10 in the *L. reuteri* FYNLJ109L1 group was 1.82 times higher than that in the HFD group (*p* < 0.01), which was similar to that in the NC mice.

### 3.4. Effect of L. reuteri on SCFAs

**Figure 5:**
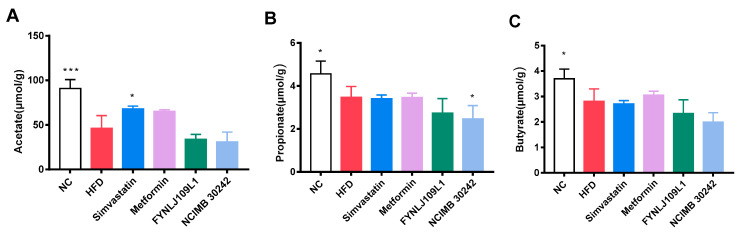
Effects of *L. reuteri* intervention on fecal SCFAs. (**A**) Acetic acid; (**B**) Propionic acid; (**C**) Butyric acid. NC, normal control; HFD, high-fat diet. Data are represented as mean ± SEM (*n* = 8). * *p* < 0.05 and *** *p* < 0.001 for comparison with HFD.

There were differences in SCFAs in the feces of mice fed with the different diets. The HFD resulted in a decrease in SCFAs in the feces, and acetic, propionic and butyric acids were significantly decreased to 49.24% (*p* < 0.001), 23.90% (*p* < 0.05) and 24.01% (*p* < 0.05), respectively (Figure 5A–C). Moreover, no significant change was found for those three acids in those mice intervented with *L. reuteri* strains, except propionic acid in the *L. reuteri* NCIMB 30,242 (*p* < 0.01).

### 3.5. Modulation of Gut Microbiota by L. reuteri

**Figure 6:**
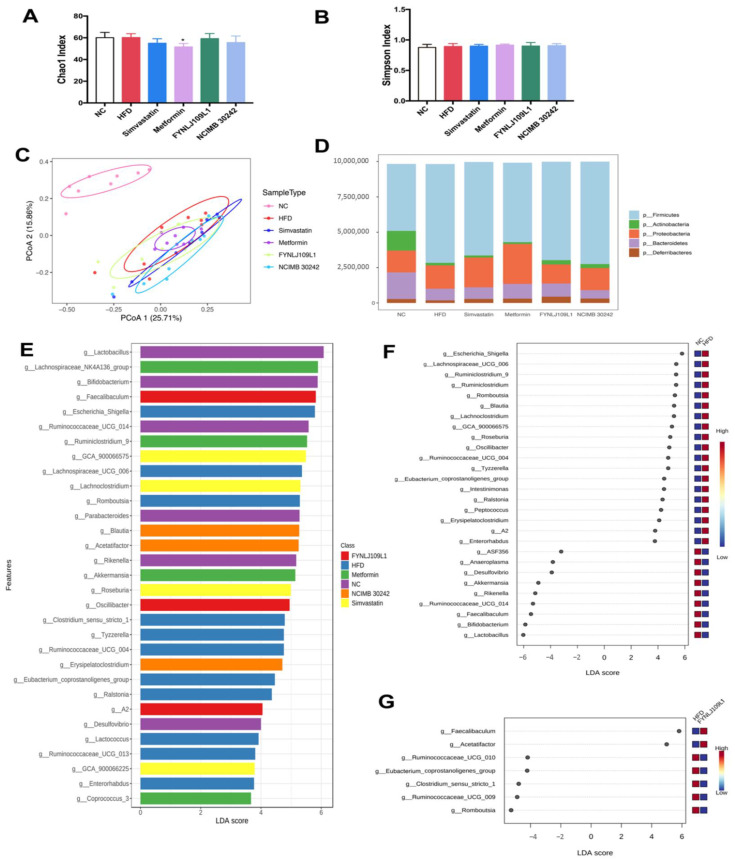
The gut microbiota modulation by the *L. reuteri* intervention. (**A**) Chao1 index; (**B**) Simpson index; (**C**) PCoA; (**D**) Microbial distribution at the phylum level; (**E**) LEfSe analysis of the gut microbiota (LDA value > 3.0); (**F**) LEfSe analysis between the NC and HFD (LDA value > 3.0); (**G**) LEfSe analysis between the HFD and FYNLJ109L1 (LDA value > 3.0). (*n* = 8. * *p* < 0.05 for comparison with the HFD).

Both diet and *L. reuteri* interventions could affect the gut microbiota. Amplicon sequencing was performed to analyze the changes in the gut microbiota in terms of diversity and composition. Chao1, which represents a species abundance, and Simpson index, which represents the microbial diversity, were used to analyze the diversity of the gut microbiota among groups. No difference was found in the Chao1 and Simpson indexes between the HFD and NC groups, indicating that the HFD had no influence on the gut microbiota diversity (Figure 6A,B). Both the *L. reuteri* strains used for the intervention also did not significantly affect the gut microbiota diversity. Beta diversity reflected in the PCoA analysis based on the Bray–Curtis distance indicated that there was a significant bacterial community between the NC group on the normal control diet and the other groups with an HFD (Figure 6C). Notably, 14-weeks of *L. reuteri* treatments in MS mice resulted in a partial normalization of intestinal microbial diversity in PCoA1 (25.70%) or PCoA2 (15.95%).

The profile of the gut microbiota changed with the different diets. At the phylum level, firmicutes (47.33%) and bacteroidetes (18.79%) accounted for the largest proportion in the normal control group, followed by proteobacteria (15.40%) and actinobacteria (13.94%). After a 14-week intake of the HFD, the proportions of the four major bacteria changed, and the relative abundance of firmicutes and proteobacteria raised to 69.85% and 16.49%, respectively, whereas the relative abundance of bacteroidetes and actinobacteria were reduced to 8.24% and 1.81%, respectively (Figure 6D). Compared with the control mice, the ratio of bacteroides to firmicutes associated with obesity in the HFD group decreased significantly (*p* < 0.01), and the increasing trend could be reversed with treatment of each *L. reuteri* strain. With the LEfSe analysis, the species with significant differences in abundance among the groups were mainly *Lactobacillus, Bifidobacterium, Ruminococcaceae UCG_014*, *Parabacteroides, Rikenella* and *Desulfovibrio* in the control group, whereas *Escherichia_Shigella*, *Clostridium_sensu_stricto_1*, *Lachnospiraceae UCG_006, Romboutsia*, *Tyzzerella, Ruminococcaceae UCG_004*, *Ralstonia, Lactococcus*, *Ruminococcaceae UCG_013*, *Eubacterium_coprostanoligenes_group* and *Enterorhabdus* were enriched in the HFD group. Moreover, *Faecalibaculum*, *Oscillibacter* and *A2* were the dominant genus in the *L. reuteri* FYNLJ109L1-treated mice (Figure 6E). Compared with the NC mice, the gut microbiota of the mice with a 14-week intake of an HFD showed an increased abundance of 19 genera and decreased the abundance of 9 genera (Figure 6F). In addition, treatments with *L. reuteri* affected 7 genera, which were *Faecalibaculum, Eubacterium_coprostanoligenes_group*, *Acetatifactor*, *Ruminococcaceae_UCG_009*, *Ruminococcaceae_UCG_010*, *Clostridium_sensu_stricto_1* and *Romboutsia* (Figure 6G).

## 4. Discussion

MS and a series of related diseases are among the global human health issues. In recent years, regulating the gut microbiota by taking probiotics has become a new approach to alleviating MS. Additionally, there is more evidence that gut microbes can produce molecules to regulate the appetite and satiety that play key roles in energy conversion and reducing the accumulation of adipose tissue [27]. For example, administration of *Lactobacillus* fermented milk in Wistar rats was shown to regulate the production of the satiety gratitude hormone [28]. Our results also confirmed that both *L. reuteri* interventions significantly decreased the daily feed intake, energy conversion efficiency and epididymal fat proportion [29]. Furthermore, the *L. reuteri* intervention in the mice fed with an HFD significantly reduced the concentration of LDL-C and the ratio of LDL-C/HDL-C, suggesting that the *L. reuteri* intervention could descrase the risk of coronary disease [30] and its related metabolic syndrome to a certain extent. Moreover, between the two strains analyzed, the mice that were fed with an HFD and treated with *L. reuteri* FYNLJ109L1 showed weaker MS symptoms, which may be due to the bacterial surface substance [31], gut microbiota modulation [32] and attenuate inflammation [33].

The liver manifestation of MS is NAFLD, which is the target organ damage caused by the disorder of blood glucose and lipid metabolism. A fatty liver occurs in patients with high body fat if the liver fat accumulation is excessive, which can be alleviated and improved through early detection and treatment [9]. NAFLD in the mice induced by a high-fructose diet can be protected by a *L. rhamnosus* GG intervention [34], and our results also reached a similar conclusion. Even the high-temperature inactivated *L. reuteri* GMNL-263 can reduce the fatty liver syndrome in HFD-fed hamsters, reduce the degree of liver fibrosis, and decrease the serum LDL-C and plasma malondialdehyde [31]. Therefore, intervention with *L. reuteri* FYNLJ109L1 nor *L. reuteri* NCIMB 30,242 alleviated those liver injury symptoms, which may be responsible for the potentially beneficial substances in the strain and needs to be verified by further high-temperature killing inactivation.

MS and related diseases can cause biological disorders such as adverse changes in intestinal bacteria [35]. Previous reports have shown that probiotic interventions can alleviate MS by regulating the gut microbiota and even rebalance those damaged by an HFD [12,33]. Among the two largest bacterial phyla with a great difference between obese and lean individuals, obese subjects had a highly relative abundance of firmicutes [36], whereas thin subjects had greater bacteroidetes in the gut, resulting in weight loss [37], which was consistent with our results. An increased abundance in firmicutes and a decreased abundance in bacteroides can lead to an increase in heat and obesity [4]. Reducing the ratio of firmicutes/bacteroidetes could reduce the energy acquisition and may have potential benefits for preventing fatty liver disease [4]. In fact, our results were in line with this, which showed with a lower ratio of firmicutes/bacteroidetes in the control compared with the HFD-fed mice and virtually no areas of steatosis in liver tissues. For the other phylum, proteobacteria represent microbial biomarkers for imbalanced gut microbial communities [38] and actinobacteria are positively associated with dietary fat intake [39]. Furthermore, in accordance with our results, proteobacteria increased in the HFD group, and it returned to normal in the mice treated with probiotics. The fact that the abundance of actinobacteria decreased in the HFD group, but increased in the control group and *L. reuteri* FYNLJ109L1 group, was puzzling. Thus, the effective intervention effect of *L. reuteri* may be due to the correlative effect of gut microbiota, but the analysis of detailed taxa still needs to be performed.

At the genus level, a long-term HFD resulted in changes in the species and abundance of the host intestinal microbes. Among the genus altered significantly, romboutsia is sensitive to bile salt and associated with energy metabolism, making it a candidate for predicting and treating the metabolic diseases [40]. Prior research showed that clostridium spp. is the causative agent of intestinal disease in goats [41], suggesting that clostridium has an adverse effect on the intestinal barrier [42]. Therefore, the accumulation of clostridium sensu stricto-1 in the gut may be responsible for the aggravation of MS symptoms in mice. The decreased abundance of these bacteria may be one of the reasons for the protective effects of *L. reuteri* FYNLJ109L1 on atherosclerosis and dyslipidemia. Moreover, acetatifactor was increased in mice fed with an HFD and treated with tea extracts [29]. Compared with the NC group, the abundance of faecalibaculum in the HFD group showed a lower relative abundance [43], and the reduced abundance of ruminococcaceae is conducive to intestinal homeostasis [44]. The reduction of SCFAs producers such as ruminococcaceae, eubacterium and faecalibacterium may lead to the reduction in SCFAs in *L. reuteri* FYNLJ109L1 treated mice. The previous literature has reported that the increase in SCFAs may alleviate the metabolic syndrome; however, even if SCFAs production is reduced, other metabolites may also be involved, such as indoles, produced by *L. reuteri* from tryptophan, which have been reported to relieve MS [1]. Therefore, at the genus level, further analysis of gut microbiota composition showed the *L. reuteri* FYNLJ109L1 intervention could effectively regulate several key taxa of an imbalanced gut microbiota with a long-term high-fat diet, and was also one of the reasons for improving MS.

In addition, an abnormal gut microbiota can promote inflammation, resulting in a state of low-grade systemic inflammation [45]. The balance of the gut microbiota is important for maintaining the normal physiological function of the intestinal barrier [46], which may otherwise lead to an increased gut permeability and LPS translocation, resulting in a subclinical proinflammatory state [47]. When endotoxins produced by the abnormal intestinal microorganisms induced TLR-4 activation by altering the gut–liver axis, this, in turn, induced the pro-inflammatory responses by activating IL-6 and TNF-α in the liver [48]. It is well known that IL-6 is one of the typical cytokines associated with insulin resistance. In particular, it can induce insulin resistance by a phosphorylation reduction of insulin receptor substrates or transcription inhibition [49]. TNF-α is an important mediator for insulin resistance in obese subjects, and it mainly induces insulin resistance by weakening the insulin receptor signal transduction [50]. Furthermore, the elevated TNF-α levels can reduce the lipid oxidation and increase the lipid accumulation, leading to obesity [51]. The main advantage of probiotics is that they can reduce the inflammation by altering the gut microbiota and alleviating the associated pathological conditions. It has been shown that some *Lactobacillus* secrete soluble molecules that inhibit the production of TNF-α via activating the mononuclear macrophages [52]. In our study, it was found that the long-term HFD increased the IL-6 and TNF-α, which were typical pro-inflammatory cytokines; moreover, administration of *L. reuteri* FYNLJ109L1 significantly inhibited the increase in TNF-α and IL-6. In addition, IL-10 is a typical anti-inflammatory cytokine which could prevent the insulin resistance and MS in mice [53]. An increase in IL-10 in probiotic-treated mice with a HFD may also contribute to ameliorating the metabolic changes, which is consistent with previous findings, connecting the changes of intestinal microbial composition with a reduction in low-grade inflammation and metabolic dysfunction [54].

## 5. Conclusions

*L. reuteri* showed a strain-dependent protective effect on metabolic disorders. *L. reuteri* FYNLJ109L1 may reduce weight gain, fat accumulation, insulin resistance, dyslipidemia, and liver tissue damage by modulating the gut microbiota (specifically via a decrease in romboutsia and clostridium sensu stricto-1 and an increase in acetatifactor).

## Figures and Tables

**Table 1:** Treatment of each group.

Group	Diet	Gavage
NC	NC	0.2 mL physiological saline + lyophilized protection agents
HFD	HFD	0.2 mL physiological saline + lyophilized protection agents
Simvastatin	HFD	0.2 mL physiological saline + 3 mg/kg/BW simvastatin + lyophilized protection agents
Metformin	HFD	0.2 mL physiological saline + 150 mg/kg/BW metformin + lyophilized protection agents
FYNLJ109L1	HFD	0.2 mL physiological saline + 5 × 10^9^ CFU/mL FYNLJ109L1 + lyophilized protection agents
NCIMB 30242	HFD	0.2 mL physiological saline + 5 × 10^9^ CFU/mL NCIMB 30,242 + lyophilized protection agents
